# Success Rate of External Dacryocystorhinostomy With and Without Stent

**DOI:** 10.7759/cureus.63683

**Published:** 2024-07-02

**Authors:** Hana Aboauf, Razan A Alawaz

**Affiliations:** 1 Ophthalmology, Ministry of Health, Riyadh, SAU; 2 Ophthalmology, Saudi Board Residency Program, Medina, SAU

**Keywords:** tubes, success rate, silicone, external dacryocystorhinostomy, dacryocystorhinostomy

## Abstract

Dacryocystorhinostomy (DCR) is a procedure that bypasses an obstruction in the nasolacrimal duct system. DCR can be categorized into two primary techniques: external and endoscopic. This review aims to assess the success rate of external DCR procedures, both with and without the use of a stent.

This study compared the outcomes of primary DCR with and without silicone intubation in multiple studies. The reviewed studies consistently showed that silicone intubation significantly improves the success rates of DCR, with success rates ranging from 80% to 95%. The involved studies suggest that silicone intubation offers specific advantages in complex cases involving distant and common canaliculus blockages and recurrent DCR surgeries.

This review emphasizes that multiple studies have documented higher success rates of external dacryocystorhinostomy (DCR) when silicone tubes are utilized as stents. Furthermore, external DCR has been identified as the preferred procedure among medical professionals.

## Introduction and background

Dacryocystorhinostomy (DCR) is a procedure that bypasses an obstruction in the nasolacrimal duct system. This was performed by creating a 1-2 cm incision on the side of the nose and removing a small piece of bone, resulting in an additional pathway for tears to drain down the nose. The procedure is carried out on adults and children with chronic epiphora and dacryocystitis caused by a total or partial nasolacrimal duct obstruction [1,2]. DCR was classified into two basic techniques: external and endoscopic, with numerous procedures reported such as laser-supported endoscopic DCR and the use of various silicone stents.

External DCR is the most commonly performed operation for obstructed tear ducts, with a success rate of more than 90%. This approach was first published in 1904 and later modified by stitching the nasal and lacrimal mucosal flaps to generate an epithelium-lined fistula [3]. A small incision is made on the side of the nose, and a small amount of bone between the tear sac and the nose is removed. Flexible tubes are used to keep the new passage open. The tube is typically removed within a few weeks or months, based on the surgeon's recommendations. This treatment is typically administered under general anesthesia and may need a longer recovery period than endoscopic DCR [4]. Endoscopic dacryocystorhinostomy is a known treatment for epiphora caused by a structural or functional blockage of the nasolacrimal duct. Endoscopic DCR is a less invasive approach that employs an endoscope to create a new drainage channel. This method is less painful than external DCR and does not require an external incision. This treatment was done by making a small incision in the nose, and an endoscope was used to find the lacrimal sac. A small drill is used to remove the bone that covers the sac, forming an ostium between the sac and the nasal cavity. Next, a stent, usually a silicone tube, is inserted from the lacrimal punctum and guides it through the canaliculus to the new orifice, reinforcing the new lacrimal drainage pathway and promoting healing. Endoscopic DCR can be done under general or local anesthesia with sedation.

The majority of physicians frequently intubate silicone tubes during external DCR for better results. Gibbs was the first to describe the use of silicone tubing to aid in the repair of the nasolacrimal system [5,6]. The technique was later developed and used as a step in external DCR. Intubation has been considered a way to prevent osteotomy or common canaliculus obstruction.

External DCR remains the standard for primary cases, with a success rate of 80-95%, despite the introduction of novel procedures including endoscopic DCR. This review aimed to determine the success rate of external DCR with and without a stent.

## Review

External DCR has been the most commonly used therapy for nasolacrimal duct obstruction. This method is known for its high success rate and satisfied patients. Although endoscopic DCR has gained popularity over the past decade, many physicians prefer external DCR, reporting higher success rates [7]. External DCR provides a large, unobstructed surgical area, allowing for direct and clear visibility of the lacrimal drainage canal and surrounding structures. Furthermore, the characteristics of lacrimal drainage blockage can be assessed more precisely intraoperatively following external DCR [7-9].

In a traditional external DCR procedure, the skin is incised along the side of the nose and the orbicularis muscle is cut to reveal the lacrimal sac. A surgical incision is made in the lacrimal bone to access the nasal mucosa. A permanent drainage passage is established through incisions in the lacrimal sac and nasal mucosa, which are subsequently stitched together. Modifications have been made to improve this traditional approach. Adjustments have been made to enhance this conventional method. Modern techniques involve the creation and suturing of anterior and posterior flaps to enhance the patency rates of the ostium [10]. Additionally, mitomycin C is utilized at the surgical site to minimize scarring and improve long-term success [11]. Another method is the use of a silicone tube to maintain the patency of the nasolacrimal duct [6].

Considerations for external DCR

Ullrich and colleagues (2023) presented considerations for external DCR. They reported that an external DCR is a viable choice for elderly patients who cannot tolerate the operation under general anesthesia alone. External DCR can be performed with local anesthesia and all bleeding during surgery can be readily handled. When a biopsy of the lacrimal sac is required, the external DCR may be slightly more practical. Furthermore, it may make surgery easier and more predictable for people who have had past facial fractures or have unique anatomy, as well as those who have proximal or mid-canalicular stenosis, allowing for retrograde intubation [2].

Silicone tubes in dacryocystorhinostomy

A significant advancement in lacrimal surgery is the introduction of silicone tubes [12,13]. It has been administered in conjunction with dacryocystorhinostomy to ensure the patency of the nasolacrimal duct subsequent to its excision and to prevent adhesions of nasolacrimal mucosa during the healing process.

Stents were frequently used in several studies for external and endoscopic DCR. Stents may have both functional and mechanical implications [14-16]. At a functional level, the silicone stent's surface tension allows tears to migrate around its edges. The stent also mechanically widens the lumen and straightens canalicular bends, increasing flow across these narrow channels [2]. Stents are usually silicone tubes introduced into the tear drainage system during surgery. These stents could be used in maintaining the newly developed drainage pathways. According to Rose's theory, the function of the stent is to avoid cross-adhesion due to epithelial scratching in the puncta, canaliculi, and valve of Rosenmuller resulting from the insertion of the probe. However, it can partially block the tear drainage system, causing discomfort until they are removed [17].

Success rate of external DCR versus endoscopic DCR

In terms of success rate, previous studies have shown a higher rate of success of external DCR than endoscopic DCR. In the prospective randomized comparative study of Nailwal et al. [18], the overall success rate of external dacryocystorhinostomy after three months of surgery was 93.33%, whereas that of endonasal dacryocystorhinostomy was 90%, but this difference was not statistically significant (p=1.0). Dasgupta et al. [19] reported the same findings, with an overall success rate of 92.80% for DCR surgery. The intergroup success rate was found to be nearly the same, with the Ex-DCR group having 94.54% success and the En-DCR having 91.07%. Eris et al. [20] found the success rates for endoscopic and external DCR procedures were 91.1% and 92.3%, respectively. Their data also suggested that patient satisfaction was higher with endoscopic DCR compared to external DCR since external DCR left visible scars in 17% of their patients.

Salih et al. [21] found that external DCR had a better overall success rate (82.5%) than endoscopic DCR (77.5%). They reported that external DCR had a success rate of 85% with intubation and 80% without intubation. Their findings further showed that endoscopic and external DCR with silicone intubation yielded comparable results to those without intubation, with no significant differences.

According to an Indian journal, the success of DCR surgery can be measured by anatomical or functional outcomes. Anatomical success is the objective analysis of the nasolacrimal canal's patency through syringing, dacryocystorhinostomy, and neo-ostium assessment during nasal endoscopy. Functional success is a subjective assessment of epiphora that can be quantified using surveys and questionnaires to study the reduction of symptoms [22].

Lee et al. (2017) studied 769 patients of Seoul National University Hospital and found that 760 (98.8%) achieved anatomical success and 630 (81.9%) obtained functional success after External DCR. They highlighted the important risk factor of functional failure, which was the site of obstruction while the small sac was a risk factor for those who had primary nasolacrimal duct obstruction [7].

Success rates of external DCR with silicone intubation

Several comparative studies have shown that using silicone intubation in primary DCR improved the success rate of DCR without intubation [23,24]. In the study by Rather et al. [25], the success rate for external DCR without silicone tube intubation (STI) was 80%, whereas the success rate for DCR with STI increased to 92%, showing that DCR with STI is a highly effective technique with an overall success rate of more than 90%. They also discovered that trauma and inappropriate intraoperative probing of the common canaliculus were the causes of postoperative common canalicular fibrosis.

Afzal et al. [26] showed that external dacryocystorhinostomy with silicone intubation is more successful than without silicone intubation. In their study, they discovered a success rate of 80.0% in group A (Dacryocystorhinostomy without silicone intubation) and 92.5% in group B (Dacryocystorhinostomy without silicone intubation).

The cumulative meta-analysis of Xie et al. [27] demonstrated that the success rate of DCR with silicone tubing was significantly higher than that of DCR without silicone tubing, particularly in the EX-DCR subgroup. The findings suggested that silicone intubation was effective in managing nasolacrimal duct obstruction during external DCR.

In the study of Kaçaniku and Spahiu [6], the success rate was measured using lacrimal patency to irrigation and epiphora relief. Patency was obtained in 89.9% of cases with epiphora recurring in 10.1% of cases. Patency was higher in the intubated group (95.1%) than in the non-intubated group (87.5%) (p > 0.05). Long et al. [28] studied the surgical success rate of external DCR in Hospital Selayang, Malaysia. They found a 90.5% success rate which is consistent with the worldwide success rate of external DCR about 80-95%. This may be due to the use of surgical methods including anterior suspended flap modification and posterior flap excision.

Sen et al. [29] evaluated the efficacy of external dacryocystorhinostomy (DCR) with silicone intubation in children below the age of 6. Their findings indicated that despite encountering challenges during the surgery, such as extensive adhesion around the perisac (n = 28) and significant bleeding (n = 24), a total of 82.61% of the cases achieved a favorable outcome. Hence, employing dacryocystorhinostomy (DCR) with silicone tube intubation is a safe and effective surgical in the treatment of recurrent lacrimal abscesses in children below the age of 6, who have congenital nasolacrimal duct obstruction.

Pandya and colleagues [30] reported that the patients with anatomical nasolacrimal obstruction exhibited significantly superior results in comparison to those with functional obstruction (P = 0.04). The use of silicone intubation for a duration exceeding six months was found to be correlated with improved outcomes (P = 0.002).

Afzal et al. [26] showed that the overall surgical success rate for external DCR was 90.5%, which is consistent with the worldwide success rate of external DCR about 80-95%. This may be due to the use of surgical methods, including anterior suspended flap modification and posterior flap excision. The first research that studied the long-term results of DCR surgery with only one week of intubation was conducted by Bohman and Kopp [14]. The success rate was 97% with no incidences of canalicular laceration, stent prolapse, or extrusion. Thus, quick intubation time is possible in uncomplicated cases. Hwang et al. (2009) investigated the efficacy of DCR with canaliculoplasty and double or single silicone intubation [31]. Their investigation only covered cases of common canalicular blockage. They showed functional success rates of 88.3% and 81.2% in double and single intubation, respectively.

Rather and Singh [25] conducted a study that revealed that the success rate for external DCR without silicone intubation was 80%, whereas the success rate for DCR with silicone intubation was 92%. According to their findings, the utilization of a silicone tube prevents the closure of the common canalicular aperture, which is the most frequent reason for failure. This, in turn, improves the success rate of DCR.

The meta-analysis conducted by Evereklioglu et al. (2023) could not identify any statistically significant differences among the therapies including endoscopic, external, and transcanalicular laser without silicone stent in RCTs [32]. The rank probability analysis revealed that Endonasal DCR with silicone stent treatment was the preferred surgical choice when considering patients with nasal disease. Conversely, external DCR with silicone stent was the preferred surgical option when patients with nasal disease were excluded from the analysis.

Other studies suggested that the use of silicone intubation may be done in complicated instances with distant and common canaliculus blockage, and recurrent DCR surgeries [33,34]. According to Buttanri et al. [35], during external DCR, the canalicular system should be often monitored. If distal or common canalicular blockage is identified, silicone tube intubation may be necessary. However, the silicone tube may temporarily block the canalicular system and create epiphora in patients. After removing the silicone tube, the majority of patients in the study with canalicular difficulties experience relief from epiphora.

Sodhi et al. employed intubation as a part of External DCR exclusively for patients considered to be at an elevated risk of unfavorable results. Their investigation specifically included cases that involved previous injuries to the medial canthus, unsuccessful surgeries for dacryocystorhinostomy (DCR), or acute episodes of dacryocystitis. They asserted an achievement rate of 76%. The study conducted by Elmorsy and Fayek (2009) aimed to assess the effectiveness of rubber tubes and silicone tubes in the osteotomy site of dacryocystorhinostomy through a randomized prospective design [36]. It has been discovered that using a silicone tube instead of a rubber one is more efficient in maintaining a bigger osteotomy following dacryocystorhinostomy. This can enhance the long-term efficacy of the operation.

In the most recent prospective study, the safety and efficacy of conventional external DCR were compared to external DCR using a Pawar silicone implant in chronic dacryocystitis. The study showed that implant surgery is safe, time-saving, and less traumatic than conventional DCR procedures. The smaller skin incision, the lack of need for nasal packing, shorter hospital stay, and early ambulation are obvious advantages in implant surgeries in the follow-up period. The Pawar intracystic implant gives a highly predictable result with minimal complications, making it a better alternative to a conventional external DCR [37].

Fadime and colleagues (2014) assessed the results of using a bicanalicular silicone tube stenting in external dacryocystorhinostomy [38]. Their research demonstrated that using the technique of stenting in complex cases significantly improves the success rates of dacryocystorhinostomy. Nevertheless, in complex situations, particularly when there are no anterior flaps, the use of stenting does not lead to higher success rates, in contrast to the loss of posterior flaps.

Previous research reported that intubation has the disadvantage of causing an increase in surgical costs. According to Buttarin and Serin (2014), the utilization of silicone intubation results in increased expenses and prolongs the period of postoperative observation for patients [39]. They advised utilizing silicone intubation for DCR surgery in particular instances of distal or common canalicular obstructions [39].

Saiju et al. [40] reported a high success rate in both the DCR-with and DCR-without tubes groups. Additionally, after a six-month follow-up, it was discovered that the success rates were 90% in the silicone group and 87% in the silicone-free group. The disparity between the groups was deemed statistically insignificant. They also reported that the use of a silicone tube resulted in a 20% rise in the cost of the surgery.

Although complications reported are low, there can be instances where the presence of silicone tubes may cause problems [41,42]. Complications associated with silicone intubation include punctal and canalicular lacerations, tube loss, foreign body sensation, and conjunctival irritation, which were also reported in these studies. Another point of controversy about using silicone tubes is how long the tube should be kept. Some authors suggested using it for a period of six or three months in cases of common canalicular issues or a high chance of failure [31,33].

Kim et al. conducted a study on the bacterial infection of silicone tubes that were removed from patients who had undergone DCR. They examined the relationship between the culture results and the postoperative clinical features. It was discovered that a variety of bacterial species were grown from the extracted silicone tubes. There is a significant relationship found between Pseudomonas (P.) aeruginosa infection and the blockage of nasal mucosa, extended silicone intubation, and surgical failure.

Table 1 summarizes the results of our review.

**Table 1:** Summary of results

Author	Study	Results
Rather et al. (2013) [25]	External dacryocystorhinostomy with and without silicon tube intubation in chronic dacryocystitis with nasolacrimal duct block	The success rate of Ex-DCR without STI is 80% while 92% with STI. The study found that the utilization of a silicone tube prevents the closure of the common canalicular aperture
Evereklioglu et al. (2023) [32]	The success rate of external, endonasal, and trans canalicular laser DCR with or without silicone stent intubation for NLD obstruction: a network meta-analysis of randomized controlled trials	No statistically significant differences were found among the therapies including endoscopic, external, and trans canalicular laser without silicone stent in RCTs. External DCR with silicone stent was the preferred surgical option for patients without nasal disease
Buttanri et al. (2012) [35]	The outcome of silicone intubation and tube removal in external dacryocystorhinostomy patients with distal canalicular obstruction	Silicone tube intubation is necessary if distal or common canalicular blockage is identified. Patients with canalicular difficulties experience relief from epiphora
Sodhi et al. (2020) [42]	Experience with bicanalicular intubation of the lacrimal drainage apparatus combined with conventional external dacryocystorhinostomy	Used intubation for patients with previous injuries. The success rate is 76%
Elmorsy and Fayek (2009) [36]	Rubber tube versus silicone tube at the osteotomy site in external dacryocystorhinostomy	Compare the effectiveness of rubber and silicone tubes in the osteotomy site of dacryocystorhinostomy. Silicone tubes were found to be more efficient
Mishra et al (2019) [37]	External dacryocystorhinostomy conventional surgery versus the Pawar implant: A comparative study	The Pawar intracystic implant gives a highly predictable result with minimal complications. The smaller skin incision, the lack of need for nasal packing, shorter hospital stays, and early ambulation are the advantages of using the Pawar intracystic implant.
Fadime et al (2014) [38]	Outcome of External Dacryocystorhinostomy with Bicanalicular Silicone Tube Stenting	Assessed the success of the bicanalicular silicone tube. The technique of stenting in complex cases significantly improves the success rate.
Buttarin and Serin (2014) [39]	Silicone intubation indications in external dacryocystorhinostomy	The utilization of silicone intubation results in increased expenses and prolongs the period of postoperative observation for patients. Suggest the usage of silicone tube intubation in cases of distal or common canalicular obstructions.
Saiju et al. (2008) [40]	Prospective randomized comparison of external dacryocystorhinostomy with and without silicone intubation	After 6 months of follow-up, success rates were 90% in the silicone group and 87% in the silicone-free group. No statistical difference between the two groups.
Afzal et al. (2014) [26]	To compare the success rate of external dacryocystorhinostomy with and without silicon intubation in patients of nasolacrimal duct obstruction	The success rate of 80% in Dacryocystorhinostomy without silicone intubation. While 92.5% were in group B, Dacryocystorhinostomy without silicone intubation.
Xie et al. (2017) [27]	Comparing the Success Rate of Dacryocystorhinostomy With and Without Silicone Intubation: A Trial Sequential Analysis of Randomized Control Trials	Demonstrated that the success rate of DCR with silicone tubing was significantly higher than that of DCR without silicone.
Kaçaniku and Spahiu (2009) [6]	The success rate of external dacryocystorhinostomy	The success rate was measured using patency. Patency was higher in the intubated group (95.1%) than in the non-intubated group (87.5%) (p > 0.05).
Long et al. (2019) [28]	External dacryocystorhinostomy	Found a 90.5% success rate, which may be due to the use of surgical methods.
Sen et al. (2019) [29]	Surgical Outcome of External Dacryocystorhinostomy With Silicone Intubation for Recurrent Lacrimal Abscess in Children Younger Than 6 Years	Evaluated efficacy of external DCR among children below 6 years old. A total of 82.61% of the cases achieved favorable outcomes.
Pandya et al. (2010) [30]	External dacryocystorhinostomy: assessing factors that influence outcome	The use of silicone intubation for a duration exceeding 6 months was found to be correlated with improved outcomes (P = 0.002).

## Conclusions

This review has revealed that multiple studies have indicated a higher success rate of external dacryocystorhinostomy (DCR) when stents, specifically silicone tubes, are utilized. It is advisable for healthcare providers and medical associations to develop clinical practice guidelines that offer recommendations for the use of external DCR with silicone tubes in the treatment of lacrimal system blockages.

Furthermore, this review has emphasized that external DCR is the preferred surgical procedure among physicians due to its advantages, including a spacious and unobstructed surgical area, direct and clear visualization of the lacrimal drainage canal, and ease of surgery for individuals with previous facial fractures. Collaboration among ophthalmologists and other healthcare professionals involved in the management of lacrimal system issues is essential for delivering comprehensive patient care.
